# Mammalian Display Platform for the Maturation of Bispecific TCR-Based Molecules

**DOI:** 10.3390/antib11020034

**Published:** 2022-05-10

**Authors:** Janine Dilchert, Martin Hofmann, Felix Unverdorben, Roland Kontermann, Sebastian Bunk

**Affiliations:** 1 Immatics Biotechnologies GmbH, Paul-Ehrlich-Str. 15, 72076 Tuebingen, Germany; janine.dilchert@immatics.com (J.D.); martin.hofmann@immatics.com (M.H.); felix.unverdorben@immatics.com (F.U.); 2 Institute of Cell Biology and Immunology, University of Stuttgart, Allmandring 31, 70569 Stuttgart, Germany; roland.kontermann@izi.uni-stuttgart.de

**Keywords:** mammalian display, CHO, RMCE, TCR, bispecific, maturation

## Abstract

Bispecific T cell receptor (TCR)-based molecules capable of redirecting and activating T cells towards tumor cells represent a novel and promising class of biotherapeutics for the treatment of cancer. Usage of TCRs allows for targeting of intracellularly expressed and highly selective cancer antigens, but also requires a complex maturation process to increase the naturally low affinity and stability of TCRs. Even though TCR domains can be matured via phage and yeast display, these techniques share the disadvantages of non-human glycosylation patterns and the need for a later reformatting into the final bispecific format. Here, we describe the development and application of a Chinese Hamster Ovary (CHO) display for affinity engineering of TCRs in the context of the final bispecific TCR format. The recombinase-mediated cassette exchange (RCME)-based system allows for stable, single-copy integration of bispecific TCR molecules with high efficiency into a defined genetic locus of CHO cells. We used the system to isolate affinity-increased variants of bispecific T cell engaging receptor (TCER) molecules from a library encoding different CDR variants of a model TCR targeting preferentially expressed antigen in melanoma (PRAME). When expressed as a soluble protein, the selected TCER molecules exhibited strong reactivity against PRAME-positive tumor cells associated with a pronounced cytokine release from activated T cells. The obtained data support the usage of the CHO display-based maturation system for TCR affinity maturation in the context of the final bispecific TCER format.

## 1. Introduction

In oncology, the main goal of immunotherapy is to use the power of patient’s own immune cells to fight cancer [1]. Bispecific antibodies have been developed in different therapeutic areas with a main usage of retargeting T cells to cancer cells to induce T cell activation and tumor cell lysis [2]. Although this concept of bispecific T cell engagers was already proposed in the mid-1980s [3], it took more than 20 years until market approval of the first T cell retargeting bispecific molecules with catumaxomab (EpCAM × CD3) in 2009 by the European Union and blinatumomab five years later (CD19 × CD3) [2]. Notably, the first T cell receptor (TCR)-based bispecific molecule, a gp100 × CD3 ImmTAC molecule, was recently approved for the treatment of metastatic uveal melanoma [4]. Even though many clinical trials investigating bispecific antibody therapies were registered since the first approval [5], a large number of patients still do not benefit from these efforts [6], which is often either related to the lack of effective therapeutics or the lack of selective cancer targets [7,8]. TCR-based therapeutics have the potential to overcome the shortcomings of antibody-based bispecifics, which naturally bind only towards cancer antigens expressed on the cell surface, by targeting the very relevant proportion of cancer antigens expressed only intracellularly. TCRs bind to proteolyzed intra- and extracellular peptides presented in the context of the human leukocyte antigen complex (HLA) [9] and their therapeutic potential can be exploited mainly in two different ways, adoptive T cell therapies [10] and soluble bispecific TCR molecules [11]. The generation of bispecific TCR molecules requires a complex affinity maturation to increase the naturally low affinity of TCRs (1–100 µM) by several orders of magnitude. Furthermore, maturation may also be required to increase solubility and stability of the TCRs to prevent potential issues related to insolubility and high aggregation propensity [12,13,14]. Phage and yeast display systems have been successfully used for TCR maturation. However, their inability to adequately reflect human post-translational modifications, such as glycosylation, can be of disadvantage for maturation of TCRs constituting glycoproteins by nature. This aspect might even be more relevant if later production of the TCR-based bispecifics is performed in mammalian expression hosts [15,16]. For improving the maturation process of TCR-based therapeutics, we developed a maturation platform based on Chinese Hamster Ovary (CHO) cells allowing TCR maturation in the context of the final bispecific TCR format. The established system is based on a stable CHO cell line with a high expression landing pad in which biomolecules can be integrated by recombinase-mediated cassette exchange (RMCE). RMCE enables the efficient and directed insertion of a DNA fragment into a single copy, predefined locus flanked with Flp recombination target (FRT) sites for Flp recombinase recognition [17]. Using a model TCR targeting an HLA-A*02-presented peptide derived from preferentially expressed antigen in melanoma (PRAME), we demonstrated that the CHO-based system can be employed for TCR maturation in the context of the bispecific T cell engaging receptor (TCER) format. Therefore, a CHO library of TCER molecules was generated comprising thousands of TCR variants encoding different combinations of previously identified complementarity determining region (CDR) mutations in all six CDRs of the model PRAME TCR. Our system allows for simultaneous selection for target binding and counterselection for off-target binding, thereby preserving the binding specificity of the TCR during the affinity maturation process. From the selected TCER candidates we characterized 10 candidates in more detail to demonstrate their ability to recognize PRAME peptide HLA (pHLA) at a physiologically relevant level. The candidates showed strong induction of anti-tumor cell responses as measured by lysis of tumor cell lines and a robust activation of T cells supporting the use of the CHO display system for targeted engineering of bispecific TCR-based molecules.

## 2. Materials and Methods

### 2.1. Cell Culture

CHO-S cells [18] (Thermo Fisher Scientific, Waltham, MA, USA) were cultured in ActiPro (Cytiva, Marlborough, MA, USA, #SH31039.02) with 1 × HT (Gibco by Thermo Fisher Scientific, Waltham, MA, USA, #11067030) and 8 nM GlutaMax (Gibco, #35050061). A total of 1 mg/mL Geneticin (Gibco, #10131027) was added to apply selection pressure. CHO cells were cultured in suspension in a 50 mm orbital shaker at 95 rpm (flasks) or 145 rpm (50 mL cell reactors) with 5% CO_2_, 37 °C, and 70% relative humidity (rH). Cells were subcultured every 2–3 days.

After single cell sort CHO cells were cultured in 100 µL CD CHO (Gibco, #10743029) with 1:20 InstiGRO CHOPLUS (SAL Scientific Ltd, Fordingbridge, UK., #RS-1225) in a 96-well flat bottom plate and incubated for 10–14 days with a gas permeable adhesive film at 37 °C, 5% CO_2_, and 80% rH without shaking.

All tumor cell lines were cultured at 5% CO_2_, 37 °C, and 80% rH and were passaged at a confluency above 80% (adherent) or every 2–3 days (suspension). Adherent cells were detached using 0.05% Trypsin/EDTA (Gibco, #11580626) or Accutase (PromoCell, Heidelberg, Germany, #C-41310), centrifuged, and resuspended in new culture medium. Suspension cells were centrifuged and transferred into new medium. UACC257 cells [19] were cultured in RPMI1640 (Gibco, #A10491-01) supplemented with 15% FCS (Life Technologies by Thermo Fisher Scientific, Carlsbad, CA, USA, #10270106). DMEM high glucose medium (Gibco, #31966-021) with 10% FCS and 1 × MEM NEAA (Gibco, #11140050) was used for HS695T cells [20]. A375 cells [21] required DMEM high glucose medium with 10% FCS. SET-2 cells [22] were cultured in RPMI1640 and 20% FCS.

### 2.2. PBMC Isolation and Freezing

Peripheral blood mononuclear cells (PBMCs) from HLA-A*02-positive healthy donors were isolated from leukapheresis products obtained from the DRK Mannheim. In brief, blood samples were directly diluted 1:1 with DPBS (Gibco, #14190250) and carefully layered over 15 mL of Pancoll (PAN Biotech GmbH, Aidenbach, Germany, #P04-60500) and centrifuged for 20 min at room temperature (RT) at 800× *g* without brake. The obtained PBMC layer was washed with DPBS by centrifugation (300× *g*, RT, 10 min). The cell pellet was resuspended in DPBS with 1% human serum albumin (HSA) (OctaPharma, Lachen, Switzerland, #PZN-00200331) and cells were counted using the ViCell XR device (Beckmann Coulter, Brea, CA, USA). For freezing, the supernatant was discarded and cells were resuspended in RPMI1640, 11.5% HSA. RPMI1640, 11.5% HSA, 20% DMSO (WAK Chemie Medical GmbH, Steinbach, Germany, #WAK-DMSO-70) was added dropwise (*v*/*v* 1:1) directly before freezing cells in a CellCool FTS30 container and stored at −80 °C prior to transferring the cells into liquid nitrogen. PBMCs were thawed in a water bath at 37 °C. Cell suspension was transferred into CTL-Wash buffer (C.T.L. Europe, Bonn, Germany, #CTLW-10) supplemented with 0.1% L-Glutamine (Gibco, #25030-024) and 50 U/mL Benzonase (VWR International, Radnor, PA, USA, #1.01695.0001) before centrifugation and resuspension in RPMI GlutaMax (Gibco, #72400021) with 10% human serum (heat inactivated) (c.c. pro, Oberdorla, Germany), 1% Penicillin/Streptomycin (Biozym, Hessisch Oldendorf, Germany, #DE17-602), 20 µg/mL Gentamycin (Lonza, Basel, Switzerland, #17-15197), and 1% sodium pyruvate (c.c. pro, #Z-20-M) supplemented with 10 U/mL Interleukin-2 (IL-2).

### 2.3. Functional Assays

Adherent target cells were plated one day before the co-culture start in a 96-well flat bottom plate. For even distribution, plates were shaken for 30 s at 450 rpm on an orbital shaker and incubated afterwards at 37 °C, 5% CO_2_, and 80% rH in the respective cell line medium. Effector cells were thawed at the same day. The next day, medium from the adherent target cell line was removed and 50 µL/well assay medium (RPMI 1640 without phenol red (Gibco, #11835-063), 10% heat-inactivated human serum (1 × GlutaMax (Gibco, #35050-038), 1 × Penicillin/Streptomycin) supplemented with 10 U/mL IL-2 was added. Suspension cells were seeded at the day of the co-culture start. Cells were harvested by centrifugation at 300× *g* for 5 min and resuspended in the assay medium. Suspension cells were seeded into 96-well round bottom plates. Serial dilutions of TCER molecules were performed in the assay medium for final concentrations of 10 nM–0.01 pM. Effector cells were harvested and resuspended in the assay medium and added to each well. An effector-to-target ratio (E:T) of 10:1 was applied for all co-cultures. After 48 h incubation, the supernatant was either used for the assessment of target cell killing or for the measurement of cytokine levels. The CytoTox 96 non-radioactive cytotoxicity assay kit (Promega, Madison, WI, USA #G1780) was used to measure the ability of the TCER molecules to mediate lysis of tumor cells. The experiments were performed following the manufacturer’s instructions. The readout was executed using a SpectraMax i3x (Molecular devices, San José, CA, USA). Cytokine release assays were performed using the MACSPlex cytotoxicity T/NK cell kit (Miltenyi Biotec, Bergisch Galdbach, Germany, #130-125-800) following the manufacturer’s instruction. Analysis was performed with the MACSQuant X using the express mode in the MACSQuantify Software (version 2.13.2) (Miltenyi Biotec). A CD3 antibody (BioLegend, San Diego, CA, USA, #317301) and a CD28 antibody (University of Tuebingen) were used for the positive controls.

### 2.4. Stable CHO Cell Line Generation

CHO cells were seeded one day before the electroporation at a density of 1.5 × 10^6^ cells/mL. For electroporation, the cells were harvested and washed once with MaxCyte electroporation buffer. After the washing step, cells were resuspended in MaxCyte buffer at a density of 2 × 10^8^ cells/mL and pJD1_GFP vector DNA was added at final concentration of 1 µg/1 × 10^6^ cells following linearization via PvuI. The transfection was performed using an MaxCyte STx system (MaxCyte, Gaithersburg, MD, USA) using the CHO-2 program. After resting for 40 min at 37 °C, 5% CO_2_ without shaking, the cells were resuspended in culture medium at a density of 4 × 10^6^ cells/mL. The next day, cells were split to a density of 0.25 × 10^6^ cells/mL in culture medium with 1 mg/mL Geneticin. Cells were selected for 2 weeks for stable GFP expression and the top 2% GFP-expressing cells were sorted twice after the expansion of stable clones using an MA800 device (Sony, San José, CA, USA). High GFP expressing cells underwent an RMCE step exchanging GFP for RFP. Therefore, the cells were treated as described above and the pJD1_RFP donor vector (1 µg/1 × 10^6^ cells) and the Flp recombinase supplied as RNA (Miltenyi Biotec, #130-106-769) (4 µg/1 × 10^6^ cells) were added prior to electroporation. Transfected cells were sorted for GFP-negative/RFP-positive cells and single-cell sorting was performed to screen for stable single-copy integration. Single-cell clones were sent for targeted locus amplification (Cergentis, Utrecht, the Netherlands) and an appropriate clone was expanded as the new landing pad containing CHO cell line.

### 2.5. TCER Library Generation and Affinity Maturation

CDR variants were obtained from a previous yeast display maturation campaign. Separate CDRs were combined in a single library, which was ordered from GeneArt (Regensburg, Germany). Expression of each chain was controlled by an hCMV promotor. TCER library was generated by transfection of the library DNA in combination with the Flp-encoding RNA. The TCER expressing cells were sorted and expanded. Therefore, the first selection round was performed with 100 nM biotinylated PRAME pHLA (pHLA complexes were prepared as described in Section 2.6.3, below) followed by staining with Streptavidin-APC (Invitrogen by Thermo Fisher Scientific, Waltham, MA, USA, #S868). The second selection round was performed with 31.6 nM PRAME pHLA and 10 nM each of 11 UV-exchanged similar peptide-HLA tetramers, coupled to PE (TET-PE). Finally, single-cell sorting was performed using 10 nM PRAME pHLA and 10 nM of each similar pHLA TET-PE. For sorting an MA800 was used in targeted mode, and for the analyses an SA3800 (Sony, San José, CA, USA) was used.

For staining, cells were washed once in DPBS, 1% BSA, and 2 nM EDTA (Carl Roth GmbH, Karlsruhe, Germany, #8040.3) and incubated in the first staining solution for 30 min in the dark at 4 °C, washed twice, and were again incubated for 30 min in the dark at 4 °C with the second staining solution. Before measurements cells were washed once again and resuspended in DPBS, 1% BSA, and 2 nM EDTA.

Genomic information of single clones was extracted using the Phire Tissue Direct PCR Master Mix (Thermo Fisher Scientific, #F170L) following the instructions for the Dilution & Storage protocol of the manufacturer. The amplification was performed using the primers JD061 (5′-GACACGAAGCTGGCTAG-3′) and JD065 (5′-CTTGCTGGCAGAAGTAGG-3′) for the alpha chain CDRs of the TCR and JD057 (5′-CAGCGCCTACTCTGAGG-3′) and JD059 (5′-GCTGGATATCTGCAGAATTCC-3′) for the beta chain. Amplicons were sent to Microsynth Seqlab GmbH (Goettingen, Germany) for sequencing.

### 2.6. Plasmid Preparation

#### 2.6.1. Vectors

Different plasmids were created for the generation of a stable CHO cell line expressing a gene of interest from a stably integrated landing pad. The GFP and RFP containing vectors for the landing pad integration were obtained via digestion of pJD1 (in-house construct with standard genetic elements) with NheI (New England Biolabs, Ipswich, MA, USA, #R313M) as well as EcoRI (New England Biolabs, #R3101M) and subsequent cloning resulting in pJD1_GFP and pJD1_RFP, respectively. The pJD1 vector contains FRT and FRT F3 site to allow for an Flp-based exchange of the landing pad. The vector and the GFP and RFP inserts were synthesized by Genscript (Piscataway Township, NJ, USA).

#### 2.6.2. Transformation and Plasmid Isolation

For the transformation One Shot TOP10 electrocompetent *Escherichia coli* (*E. coli*) cells (Thermo Fisher Scientific, #C404052) were used following the manufacturer’s instructions. After incubation at 37 °C for 24 h, single clones were picked and expanded in LB broth (Carl Roth, #X964.1) with 100 µg/mL Ampicillin (Carl Roth, #K029.1). The NucleoBond Xtra Midi (Macherey-Nagel, Dueren, Germany, #740422.50) or Maxi EF Kit (Macherey-Nagel, #740424.50) were used for plasmid DNA isolation following the manufacturer’s instructions. Isolated DNA was heated for 20 min at 95 °C and handled sterile afterwards. A NanoDrop 8000 (Thermo Fisher Scientific) was used to determine the DNA concentrations via absorbance measurement at 260 nm.

#### 2.6.3. Soluble TCER Expression

Transient transfection was performed using the CHO program of the MaxCyte STx system. The TCER chains were supplied as separate DNA constructs cloned into expression vectors pMH1 (in-house construct) at a 1:1 ratio with 1.5 µg/1 × 10^6^ cells. Cells were washed once with the MaxCyte electroporation buffer. Cells and DNA were mixed and transferred into an OC-400 cuvette. Afterwards, cells transfected with the same construct were pooled and rested in a 25 cm^2^ cell culture flask at 37 °C, 70% rH, and 5% CO_2_. After resting, cells were transferred into a shake flask at a density of 4 × 10^6^ cells/mL. After 24 h of incubation, temperature was reduced from 37 °C to 32 °C and sodium butyrate (Sigma Aldrich, St. Louis, MO, USA, #B5887) was added to a final concentration of 1 mM. Feeding of the cultures occurred at day 4, 6, and 8 with Cell Boost 7A (Cytiva, #SH31119.01) and 7B (Cytiva, #SH31120.01), 5% and 0.5%, respectively. Cells were harvested either at day 11 or when the viability dropped under 70%. Cell supernatants were filtered using SartoClear Dynamics Lab V kits (Sartorius, Goettingen, Germany, #SDLV-0500-20E0-E). A final concentration of 0.1% sodium azide (Merck, Darmstadt, Germany, #1.06688.0025) was added and supernatants were stored at 4 °C until purification. Soluble-expressed TCER molecules were purified using tandem purification with a ProteinL and size-exclusion column installed in an Akta Pure 25 system (Cytiva). After elution of Protein L-bound protein at pH 2.8, the protein was directly applied onto a Superdex 200 pg column equilibrated in DPBS. The eluting fractions containing monomeric TCER molecules were collected and pooled. For soluble expression of the parental TCR, a single chain TCR construct containing four stabilizing TCR framework mutations was generated and expressed in fusion with the N-terminus (heavy chain) of a Fab fragment of the UCHT1 anti-CD3 antibody. Protein concentration was determined using a NanoDrop 8000 and adjusted to 1 mg/mL either by using Amicon Ultra-15 centrifugal concentrators or by dilution with DPBS. Final protein concentration was determined after 0.22 µm filtration.

### 2.7. Generation of pHLA Complexes

HLA molecules were produced in *E. coli* as inclusion bodies, purified, and refolded essentially as described by Garboczi et al. [23]. The β2m molecules and the HLA chains were transferred from the HLA injection into the refolding buffer via a syringe. In the next step, PRAME or a UV-light sensitive peptide was added with a final concentration of 30 µM. This reaction stirred for 2–4 days at 10 °C using ultrafiltration stirred cells with a 30 kDa membrane. Further purification was performed via SEC chromatography with TSBA buffer (DPBS, 2% FCS, 2 mM EDTA, 0.01% sodium azide) and a HiLoad 26/600 75 pg column in an AKTA Prime plus. Protease inhibitor PSMF (Sigma Aldrich, #P7625), leupeptin (Roche, Basel, Switzerland, #11017101001), and pepstatin (Roche, #11359053001) were added. The mixture was concentrated to 2000 µg/mL via an Amicon Ultra-15 centrifugation unit. The biotinylation process was conducted overnight via BirA biotin-protein ligase (Avidity LCC, Aurora, CO, USA, #Bulk-BirA) at 27 °C following the manufacturer’s instruction. The pHLA complexes were again gel-filtered, concentrated, and aliquoted.

All target and off-target peptides (Table 1) used were produced in-house via a standard Fmoc chemistry with a Syro II synthesizer (Biotage, Uppsala, Sweden). HPLC analyses were performed to determine the purity of the peptides. UV-light sensitive peptides were built with a 2-nitrophenylamino acid reside. Before usage peptides were solved in 10% DMSO, 0.5% TFA (VWR, #1.08262.0025) at a final concentration of 10 mg/mL and stored at −20 °C until further handling.

The UV-exchange of UV-light sensitive peptides was done as described in Rodenko et al. [24]. The peptides were incubated for 1 h at 366 nm UV-light with a biotinylated UV-light sensitive pHLA complex at a molar ratio of 100:1. Obtained biotinylated UV-exchanged pHLA complexes were tetramerized with PE-coupled streptavidin (Invitrogen, #12-4317-87) in a 4:1 molar ratio. The calculated streptavidin-PE amount was added in three separate steps with a 30 min incubation period in the dark at 4 °C and 1500 rpm in a thermomixer C (Eppendorf, Hamburg, Germany). After that, Biotin was added at a final concentration of 25 µM.

### 2.8. Biolayer Interferometry

An Octet HTX system (Sartorius, Goettingen, Germany) was used for the affinity measurements of soluble TCER molecules towards their target pHLA complexes. Kinetic binding analysis was performed using kinetics buffer (DPBS, 0.1% BSA, and 0.05% Tween-20 (Sigma Aldrich, #P1379-100ML)) to dilute all analytes to their final concentrations. Samples were loaded and measured in a 384 tilted well plate containing 60 µL of sample volume at a 3 mm sensor off-set. The used HIS1K biosensors were hydrated with kinetics buffer for at least 10 min. For the measurements the plate was kept at a temperature of 30 °C and shaken at 1000 rpm. Baselines before association phases and the following dissociation phases were performed in the same wells to enable inter-step correction. All obtained data were analyzed using the Data Analysis HT software (version 12.0). Alignment of the raw sensor data at the Y axis was performed by adjusting the data to the end of the baseline step. Inter-step correction was applied for the alignment of the dissociation phase start to the end of the association phase. Savitzky–Golay filtering was applied to smooth data. Finally, fitting of the sensograms to a 1:1 Langmuir kinetics binding model was done.

### 2.9. Data Analysis

Flow cytometry data were analyzed using the FlowJo 10.7 software (Becton Dickinson, Franklin Lake, NJ, USA).

Statistical analysis and data plotting was performed via GraphPad Prism 9.2.0 (GraphPad Prism Software, San Diego, CA, USA). EC50 values were derived from four parameter logistic sigmoidal non-linear regression.

Sequence analysis and construct planning was done within Geneious Prime software 2020.2.5 (Biomatters Ltd., Auckland, New Zealand).

## 3. Results

### 3.1. Generation of High-Expression CHO Cells for Targeted Protein Expression

**Figure 1:**
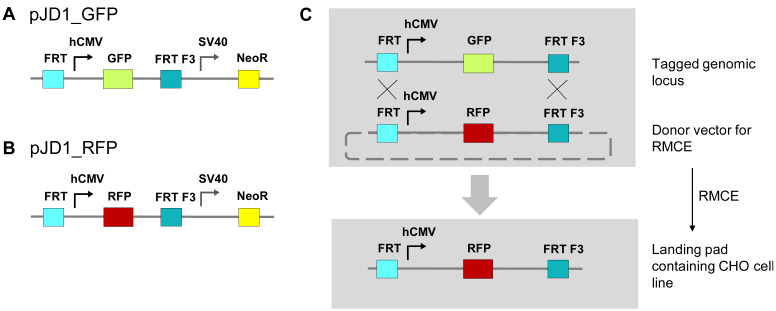
Vector design and process overview for generation of landing pad-containing CHO cell line. (**A**) pJD1_GFP vector for stable GFP integration into CHO genome. The hCMV promotor-driven GFP expression cassette is flanked by a 5′ FRT site and a 3′ FRT F3 site followed by a SV40 promotor-driven neomycin resistance cassette. (**B**) pJD1_RFP donor vector for RCME. The hCMV promotor-driven RFP expression cassette is flanked by a 5′ FRT site and a 3′ FRT F3 site followed by a SV40 promotor-driven neomycin resistance cassette. (**C**) RMCE process for generation of a landing pad-containing CHO cell line. Upon integration of the GFP-expressing landing pad into the CHO genome, an RMCE was performed to exchange it with an RFP expressing cassette. CHO cells showing only RFP expression were single-cell sorted and a stable RFP expressing clone was expanded as the landing pad containing cell line.

**Figure 2:**
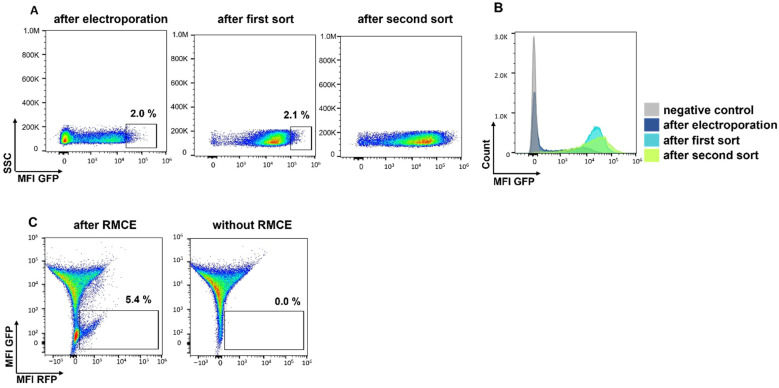
Flow cytometric analysis of CHO cells during landing pad integration. (**A**) Gating strategy of the enrichment of high expressing GFP cells. The top 2% of the GFP expressing cells were sorted two times. (**B**) The histogram plot indicates the GFP expression levels in comparison to non-transfected CHO cells (negative control) (**C**) GFP and RFP expression of CHO cells subjected to RMCE with pJD1_RFP donor vector and RNA-encoded Flp recombinase. RFP-positive/GFP-negative cells were sorted and single clones were generated for analysis of the integration site.

**Figure 3:**
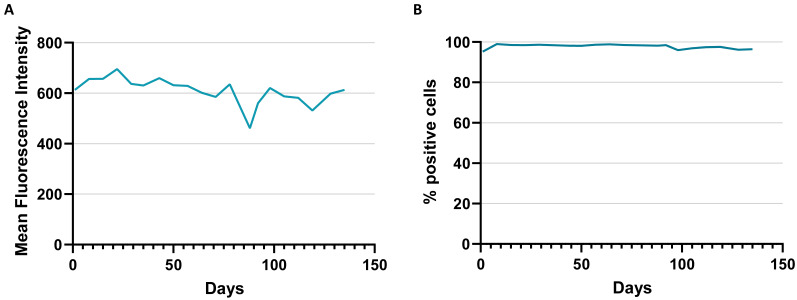
Stability of RFP expression of CHO clone RFP_A03 selected for landing pad containing CHO cell line. (**A**) RFP MFI values and (**B**) percentage of RFP-positive cells of RFP_A03 CHO cells during long-term culture of 135 days.

For the generation of a stable, high expression CHO cell line with an RCME-based retargetable landing pad, two vectors encoding for neomycin resistance, and FRT-flanked fluorescence markers GFP or RFP were designed (Figure 1). After electroporation of the GFP encoding vector pJD1_GFP into CHO cells and a subsequent culturing period of 14 days under Geneticin selection, about 2% of the cells with highest GFP expression were sorted by flow cytometry and the procedure was repeated including a second sort (Figure 2). The resulting CHO cells showed stable and high expression of GFP caused by a random integration of the GFP cassette into the genome. The GFP expressing cells were then electroporated with the RFP encoding vector pJD1_RFP together with RNA encoding Flp recombinase to mediate RMCE (Figure 1C). After the RCME process, 5.4% of the CHO cells showed RFP expression in absence of any GFP expression supporting a high exchange rate of the RFP cassette (Figure 2C). In the next step, the RFP-positive/GFP-negative cells were subjected to single-cell sorting and CHO clones were generated for assessing the genomic integration site of the RFP cassette by targeted locus amplification analysis. The analysis revealed a CHO clone (RFP_A03) with a single-copy integration of the RFP exchange cassette. To confirm stable, long-term expression of RFP the CHO clone, RFP_A03, was cultured for a period of 135 days in the absence of any Geneticin selection. During the long-term culture, RFP_A03 showed very stable and high expression level of the RFP fluorescence marker (Figure 3) supporting the use of this clone for targeted protein expression. Therefore, the clone RFP_A03 was expanded as the stable, landing pad-containing cell line allowing targeted expression and engineering of proteins in the CHO display system.

### 3.2. Generation of PRAME-Specific TCER Library and Selection of Candidates

**Figure 4:**
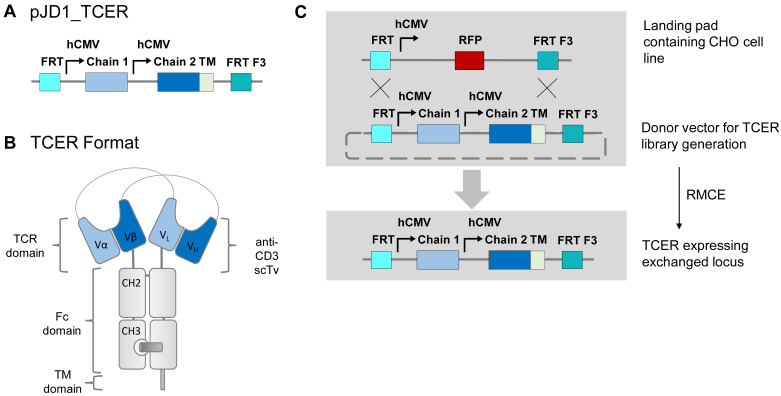
Vector design and overview of RMCE-mediated process for TCER library generation in CHO cells. (**A**) pJD1_TCER donor vector with hCMV promotor-driven expression cassettes for TCER chain 1 and 2, which are flanked by a 5′ FRT and a 3′ FRT F3 site. (**B**) Molecular structure of a TCER molecule. TCER chain 1 encodes for the variable TCR alpha chain (Vα), the variable light chain (VL) of an UCHT1 anti-CD3 antibody followed by IgG1 constant domains CH2 and CH3 modified with specific mutations to ablate Fc gamma receptor binding and complement activation as well as knob-forming mutations. The CH3 domain is followed by a PDGFR transmembrane domain for anchoring the TCER molecule on the CHO cell surface. TCER chain 2 encodes for the variable heavy chain (VH) of the anti-CD3 antibody and the variable TCR beta chain (Vb) followed by IgG1 constant domains CH2 and CH3 modified with specific mutations to ablate Fc gamma receptor binding and complement activation as well as hole-forming mutations. (**C**) RMCE process for TCER library generation in CHO cells. The RFP locus of the RFP_A03 CHO cells was exchanged via RCME with TCER containing donor vectors encoding for all potential combinations of CDR variants (see also Table 2) of a PRAME-specific model TCR.

To demonstrate that RFP-A03 CHO cells can be used for displaying and engineering of complex biomolecules such as bispecific TCR molecules, we generated a library of TCER molecules for targeted integration and expression (Figure 4). The TCER library was based on a PRAME-specific model TCR that was previously maturated in the scTv format by yeast display to increase stability and binding affinity and resulted in the identification of various variants of CDRa1, CDRa2, CDRa3, CDRb1, CDRb2, and CDRb3 of the model TCR (Table 2). The individual CDR sequences of the best performing variants of the model TCR were employed to generate a library of TCER molecules for displaying on RFP_A03 CHO cells for identifying optimal combinations of the CDR variants. The TCER library design allowed the expression of TCER molecules covering every potential combination of the individual CDRs corresponding to a minimum library size of 36,864 TCER molecules derived from 2, 6, 16, 6, 16, and 2 variants of CDRa1, CDRa2, CDRa3, CDRb1, CDRb2, and CDRb3, respectively (Table 2).

TCER molecules showing strong and specific binding to the PRAME pHLA target were selected during three rounds of CHO cell sorting according to selection and counterselection principles. The first sorting round was done with 100 nM PRAME pHLA monomer without counterselection. In a second and a third sorting round, 31.6 nM and 10 nM PRAME pHLA monomer, respectively, were used for TCER selection together with a counterselection based on 10 nM pHLA tetramers representing each of 11 different peptides being expressed on human normal tissues and showing a high degree of sequence similarity to the PRAME target peptide. From the TCER-displaying CHO cells sorted in the third round, 171 clones were sequenced resulting in 39 unique variable domain sequences of the model PRAME TCR. Based on the highest abundance of sequences and the highest strength of PRAME pHLA binding, we selected 10 TCER candidates for detailed characterization. Among the 10 selected TCER candidates, between 3 and 5 out of the 6 wild type (wt) CDRs appeared to be modified (Table 3). The most stringent effect was observed for CDRβ2, for which only 1 out of 16 input CDR variants (VHGEER) was identified indicating that this CDRβ2 variant had a major role for improving the TCER binding to PRAME pHLA. For CDRα2, CDRα3, CDRβ1, and CDRβ3, respectively, eight, nine, seven, and five variants were found among the ten selected TCER candidates. However, given that the occurrence of variants for these four CDRs cannot clearly be distinguished from a random distribution, the contribution of these CDR variants to an improved affinity can hardly be determined. For CDRα1, no variant beyond the wt was identified arguing against a contribution of this CDR in increasing TCR affinity.

**Figure 5:**
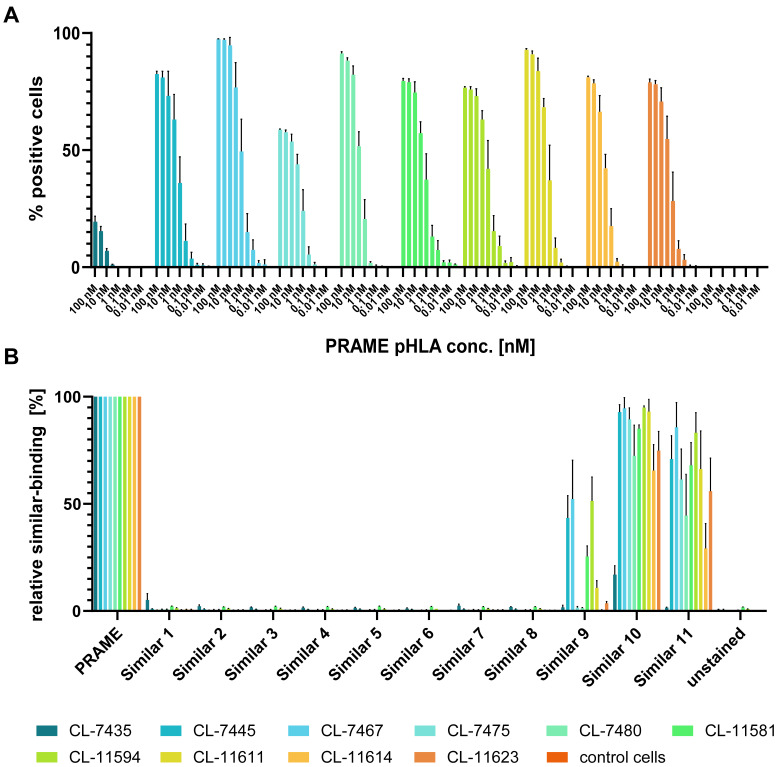
Flow-cytometric analysis of PRAME and similar peptide binding of CHO-displayed TCER candidates. (**A**) Target binding was analyzed with PRAME pHLA monomer applied at concentrations ranging from 100 nM to 10 pM, with $\sqrt{10}$ diluting steps, resulting in nine different measured concentrations. (**B**) Binding of TCER candidates to 10 nM similar peptide tetramers comprising each of the 11 different similar peptides (Table 1) in relation to the 10 nM PRAME peptide tetramer. Each datapoint represents the mean of triplicate measurements with the respective standard deviation (SD).

The selected TCER candidates (Table 3) were further assessed regarding their target binding profile. With exception of the weaker binding candidates CL-7435, CL-7475, CL-7480, and CL-11614, the TCER candidates showed strong binding to PRAME pHLA monomers with first detectable binding signals at 100 pM monomer concentration (Figure 5A). We next analyzed the binding specificity of the TCER candidates using a highly sensitive, avidity-driven analysis with pHLA tetramers, each constituting 1 of 11 different peptides with a high degree of sequence similarity to the PRAME target peptide. As shown in Figure 5B, the TCER candidates showed moderate to strong binding to 3 out of the 11 tested similar peptides. The strongest binding was observed for similar peptide 10, whose sequence is identical to the PRAME target peptide in 6 out of 9 positions (Table 1). The weakest binding out of the 3 strongly detected similar peptides was observed towards similar peptide 9 sharing 5 identical positions with the PRAME target peptide. Interestingly, moderate to high binding was also seen with similar peptide 11 sharing only the identical positions 5, 6, and 7 with the PRAME target peptide arguing for a relevant role of the c-terminal peptide stretch for binding of the TCER candidates.

### 3.3. Assessment of TCER-Mediated Anti-Tumor Activity

For functional testing, all selected TCER candidates (Table 3) were expressed in CHO cells and the purified molecules were subjected to affinity measurements using biolayer interferometry. As shown in Table 4, TCER candidates CL-7467 and CL-7445 exhibited the highest PRAME pHLA binding affinity with a KD of 3.4 and 3.7 nM, respectively. The lowest binding affinities of KD of 16.5, 17.8, 24.5, and 37.4 nM were determined for the candidates CL-7435, CL-11614, CL-7480, and CL-7475, respectively, which is in line with the lack of binding signals of these candidates observed in the CHO display system when only 0.1 nM PRAME pHLA was used for the staining (see also Figure 5A).

**Figure 6:**
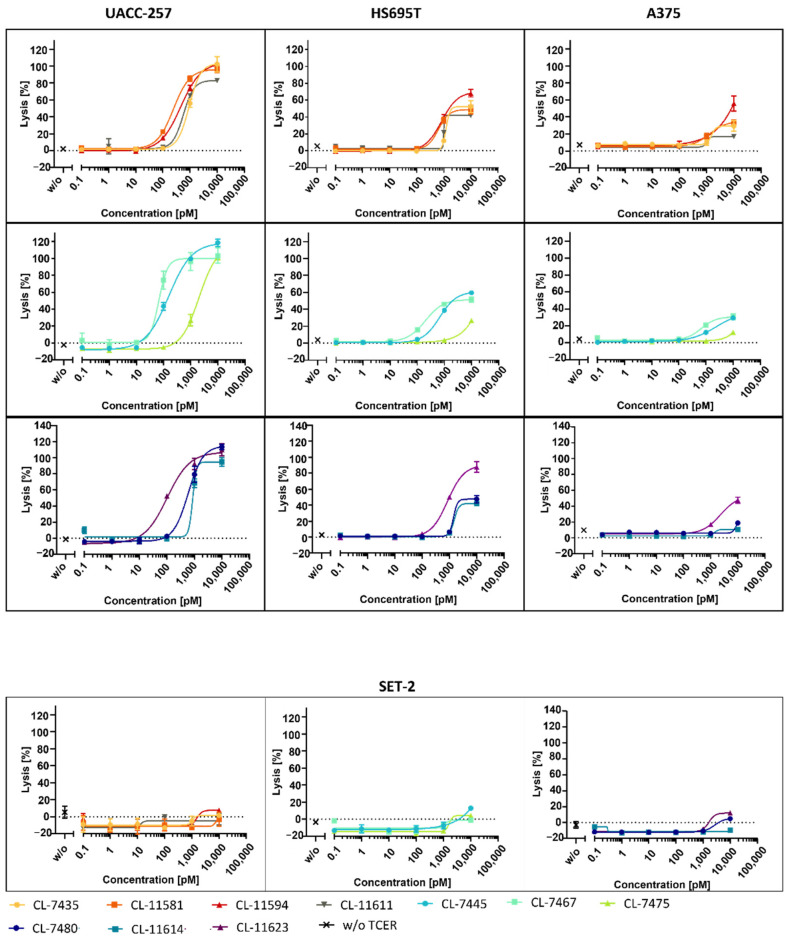
Assessment of tumor cell lysis mediated by the selected TCER candidates. PBMC of a healthy human donor were co-cultured with tumor cells at an effector-to-target ratio of 10:1 and in presence of increasing TCER concentrations. Lysis of PRAME pHLA-positive target cells UACC-257 (1100 cpc), HS695T (400–550 cpc), and A375 (50 cpc) was determined based on LDH-release assay. For control, SET-2 cells were used as a target negative cell line. Each datapoint represents the mean of triplicate measurements with the respective SD.

In the next step, we assessed the ability of the selected TCER candidates to induce PRAME peptide-dependent tumor cell lysis. LDH-release assays were performed with the target cell lines UACC-257, HS695T, and A375 presenting the PRAME pHLA at different target densities. The PRAME peptide copy numbers per cell (cpc) ranged from about 1100 cpc for UACC-257, 400–550 cpc for HS695T, and about 50 cpc for A375. As control, SET-2 cells were included in the experiments which had no detectable PRAME peptide copies on their surface. All selected TCER candidates induced target cell killing of UACC-257 and HS695T cells (Figure 6), albeit the lowest reactivity was seen with the candidates CL-7475, CL-7480, and CL-11614, in line with their lower binding affinity (Table 4). For A375 cells presenting the target PRAME peptide only at a very low density (50 cpc), weak killing activity was detectable only for TCER candidates showing higher affinity, such as CL-7467, CL-7445, CL-11581, CL-11594, and CL-11623. In line with the expectation, there was no or only very weak killing activity at the highest TCER concentration observed for all tested candidates with PRAME-negative SET-2 cells. Table 5 summarizes the EC50 values calculated from the LDH-release assays with UACC-257 and HS695T cells arguing for CL-7467 as the TCER candidate with the highest anti-tumor activity in line with its highest binding affinity.

**Figure 7:**
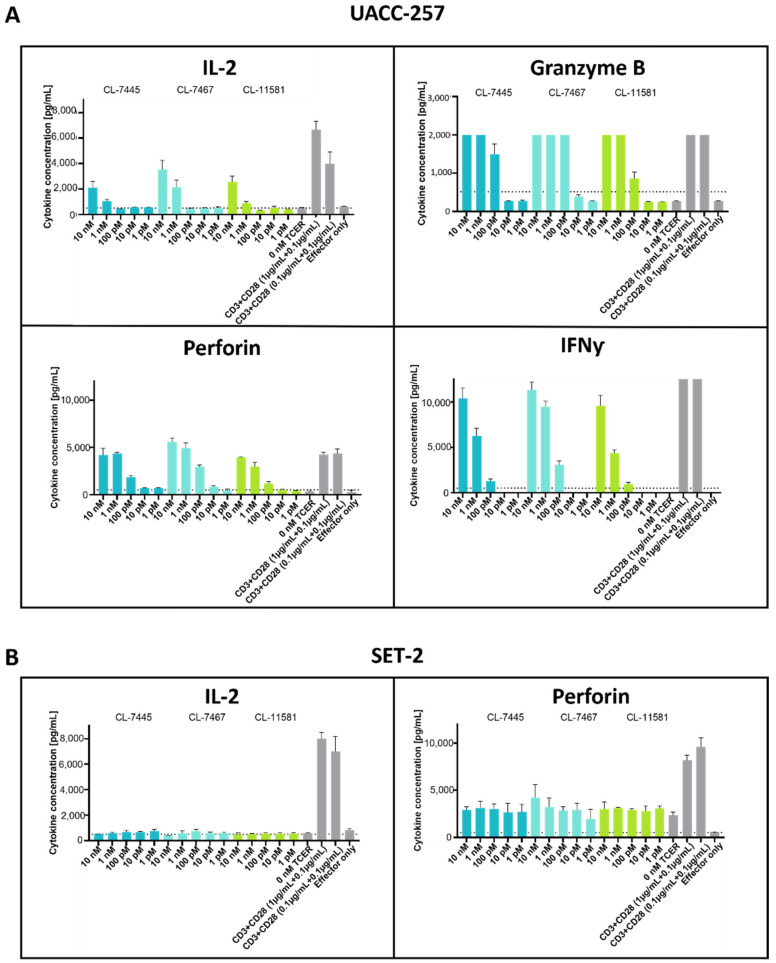
Assessment of TCER-mediated cytokine release from PBMC in response to tumor cells. PBMCs of a healthy human donor were co-cultured with tumor cells at an effector-to-target ratio of 10:1 and in presence of increasing TCER concentrations. CD3- and CD28-binding antibodies were used as positive control. Cytokine levels were determined using the MACSPlex cytotoxic T/NK cell kit. (**A**) UACC-257 (1100 cpc) was used as PRAME target pHLA-positive cell line and (**B**) SET-2 was used target negative control cell line. Each datapoint represents the mean of a triplicate with the respective SD.

We also determined cytokine levels from the killing assay co-cultures to get more insight into the immune reactions triggered by the three potent TCER candidates CL-7445, CL-7467, and CL-11581. As shown in Figure 7, the tested candidates induced release of IL-2, granzyme B, perforin, and IFNγ from human PBMC in response to UACC-257 tumor cells. Among the three candidates, CL-7467 showed highest capacity in inducing the above-mentioned cytokines, with pronounced release of perforin, granzyme B, and IFNγ already at a concentration of 100 pM TCER, while IL-2 release was pronounced at 1 nM. As expected, no induction of IL-2 and perforin release was observed for all three TCER candidates when PBMC were co-cultured with the PRAME-negative cell line SET-2.

## 4. Discussion

Here, we describe the setup and application of a mammalian display platform for targeted expression and engineering of therapeutic proteins. The platform employs CHO cells genetically modified to contain a landing pad allowing for RCME-mediated integration of genetic elements encoding for biomolecules. Genetic information integrated into CHO cells by this process will be expressed from a single copy at the same genetic locus. Such a targeted single-copy integration has significant advantages over display systems based on standard transfection, viral transduction, and transposases, which often result in incorporation of multiple copies at different integration sites [25,26,27], leading to variations in the protein expression levels making subsequent screening and selection processes complicated [28]. To facilitate the exchange efficiency, we applied an electroporation protocol in which RNA encoded Flp recombinase together with the DNA encoded donor vector was used for CHO cell transfection. This resulted in exchange rates of about 5% and compares favorably with reported exchange efficiencies achieved with alternative technologies designed for targeted protein engineering, such as CRISPR/Cas9 and TALEN, showing also up to 5% exchange efficiency [29,30]. In addition, by using an enhanced Flp version, the exchange efficiency rates may even be increased to more than 7% [31]. Thus, in combination with a MaxCyte flow electroporation system that enables electroporation of up to 1 × 10^11^ cells in a 30-min cycle, the above exchange rates would allow generation of large mammalian display libraries with a theoretical size of about 1 × 10^9^ [29]. In our study, we worked with a small library of 10^4^–10^5^ with the main goal to demonstrate usage of the generated CHO display system for engineering TCR domains in context of the full-length, bispecific TCER format. Although TCR variable domains have been affinity maturated by means of other display methods, such as phage display and yeast display [32,33,34], to our knowledge, this is the first report demonstrating feasibility of TCR engineering in the context of a final bispecific TCR format. Since CHO cells tend to express mutations identified from a CHO-based system better than mutations found in other host cells [35], the use of CHO cells for TCR engineering may have benefits for later production of the molecules in CHO cells. In addition, the usage of the final TCER format makes subsequent reformatting into the therapeutic format, which can be accompanied by loss of affinity or even loss of function, dispensable [36,37]. Furthermore, maturation of the TCR domains in the final bispecific format can potentially also identify beneficial mutations resulting in an increased interdomain stability between TCR and antibody variable domains of the TCER format, an aspect that is not possible with a reformatting process. Based on the measurement of RFP fluorescence intensity, we could confirm a highly stable expression of the inserted RFP cassette in CHO cells for a period of 135 days. This long-term expression stability in CHO cells outperforms other expression systems based on episomal vectors or random integration [38,39] and is more than sufficient to perform several rounds of biomolecule selection during an engineering campaign. For our engineering campaign, we selected a model TCR targeting an HLA-A*02-presented peptide derived from PRAME. The parental PRAME model TCR displaying non-modified CDRs exhibited a binding affinity of 1.2 µM (K_D_) when expressed as single chain TCR construct that harbored four stabilizing TCR framework mutations for soluble expression. In a previous maturation campaign based on yeast surface display, we selected binding-improved CDR variants of the PRAME model TCR, which were further employed for the study presented here to identify an optimal combination of the different CDR variants. Based on the previous work, we used the stabilized variable domain sequences and the different CDR variants of the PRAME model TCR to generate a TCER library in CHO cells, allowing for a systematic combination of all identified CDR variants to select PRAME TCER candidates with improved binding characteristics. We identified 39 unique PRAME TCR sequences, each displaying different combinations of the input CDR sequences. Interestingly, among the best binding and most frequent TCER candidates, only 1 CDRβ2 was identified out of the 16 CDRβ2 input sequences of the library supporting a successful selection process in the CHO display system, resulting in TCER variants with improved PRAME pHLA binding. We selected 10 TCER candidates for detailed assessment of PRAME pHLA binding of CHO-displayed TCER in comparison to functional anti-tumor cell responses mediated by their soluble counterparts expressed in CHO cells. In general, we observed good correlation between PRAME pHLA binding of CHO-displayed TCER and its behavior as soluble bispecific since the only four TCER candidates CL-7435, CL-7475, CL-7480, and CL-11614 lacking a CHO display binding signal with 0.1 nM PRAME pHLA also showed the weakest binding affinity in biolayer interferometry and the lowest reactivity against PRAME pHLA-positive tumor cells. This observation argues for the use of a CHO display system for predicting highly functional TCER candidates. The best TCER candidate identified in the CHO display system, CL-7467, exhibited highest binding affinity of 3.4 nM (K_D_), which is an about 400-fold increase over the parental TCR variant, and also showed highest anti-tumor cell reactivity together with the most pronounced release of perforin, granzyme B, IFNγ, and IL-2.

A prerequisite for successful TCR-based therapeutics is the ability to provoke a strong, but at the same time highly specific anti-tumor response. Thus, the assessment of unpredicted cross-reactivity is of high importance to minimize potential safety risks during clinical development. This aspect is highlighted by a severe case of cross-reactivity of an autologous TCR-T program using an affinity-enhanced TCR that targeted, besides the intended MAGE-A3 target peptide, also a similar peptide expressed in cardiomyocytes leading to fatalities in the clinical trial [40,41]. Using our CHO display system, an unintended cross-reactivity of TCER molecules can be evaluated early in the development process by testing the TCER binding towards normal tissue presented peptides with high sequence similarity to the target peptide. The binding analysis with 11 different similar peptides, whose expression on human normal tissue cells was confirmed by mass spectrometry, revealed moderate to strong binding of the selected PRAME TCER candidates towards 3 out of 11 peptides. Our observation that 8 out of 11 similar peptides did not show any binding signal strongly argues for a PRAME peptide-dependent binding of the maturated TCER variants; rather, it showed that the gain in affinity is primarily driven by increased interaction with the HLA backbone. Such an interaction would presumably translate into unspecific binding of TCER molecules to HLA-A*02 regardless of the presented peptide, and thus, would have resulted in detectable binding towards most if not all of the similar peptides tested here. Based on the sequence similarities of the three cross-reactive peptides, it can be assumed that the selected TCER candidates bind to the C-terminal stretch of the PRAME target peptide, with histidine and isoleucine at positions 5 and 7, respectively, being more relevant. In our engineering campaign, we could not identify any TCER candidate lacking binding to these off-targets despite using constant counterselection pressure during the maturation process, even though there was considerable variability in the CDR sequences of identified TCER candidates. The similar off-target binding patterns shared among the identified TCER candidates, also seen in the previous yeast display campaign, can likely be attributed to be a characteristic of the parental model TCR chosen for the maturation campaign and suggest that, for a potential therapeutic application, an alternative parental TCR with a more specific binding pattern would be required as starting point for affinity maturation. Nevertheless, the obtained data demonstrate the high value of the established CHO display system for maturation of bispecific TCR-based molecules and support its usage for the generation of pHLA-targeting TCER molecules.

## Figures and Tables

**Table 1:** Sequence information of similar peptides with “x” representing a variation to the PRAME peptide and “.” representing a wt residue.

	Sequence at Peptide Position								
	1	2	3	4	5	6	7	8	9
PRAME	S	L	L	Q	H	L	I	G	L
Similar 1	.	x	.	.	x	.	.	x	x
Similar 2	.	.	x	x	.	.	x	.	.
Similar 3	.	.	x	.	.	.	x	x	x
Similar 4	.	.	.	x	x	.	.	x	.
Similar 5	.	.	.	x	.	.	x	x	.
Similar 6	.	.	.	.	.	x	.	x	.
Similar 7	.	.	.	.	x	x	x	x	.
Similar 8	.	.	x	.	x	.	x	.	x
Similar 9	.	x	.	x	x	.	.	.	x
Similar 10	x	.	.	x	.	x	.	.	.
Similar 11	x	x	x	x	.	.	.	x	x

**Table 2:** CDR sequences for the combinatorial screening library in CHO cells.

CDRα1	CDRα2	CDRα3	CDRβ1	CDRβ2	CDRβ3
DRGSQS	YSNGDKE	DNAHGGM	SGHRS	EHGLER	CASSPWDSPNVQY
DRGSQL	YQEGDKE	DNDQGGI	EGHRA	FSETQR	CASSPWDSPNEQY
	YQTGDKE	DNDVGGI	PGHKA	IHGEER	
	YQAGDKE	DNEQGGM	PGHRA	IHGQER	
	YPQGDKK	DNKAGGI	PGHRS	IHGVER	
	YSQGDKE	DNPAGGI	QGHRA	VHGAER	
		DNPRGGM		VHGEER	
		DNPVGGP		VHGIER	
		ENKPGGP		VHGKER	
		GNAQGGM		VHGLER	
		GNDLGGI		VHGMER	
		NNPSGGM		VHGNER	
		PNPPGGK		VHGQER	
		PNTHGGP		VHGRER	
		SNFGNEK		VHGVER	
		TNIAGGS		VHGYAR	

**Table 3:** CDR sequences of selected TCER candidates from the CDR combinatorial screening in comparison to the wild type CDRs of the parental TCR.

Clone	CDRα1	CDRα2	CDRα3	CDRβ1	CDRβ2	CDRβ3
Parental TCR	DRGSQS	YSNGDKE	SNFGNEK	SGHRS	FSETGR	CASSPWDSPNEQY
CL-7435	DRGSQS	YQAGDKE	GNDLGGI	SGHRS	VHGEER	CASSPWDSPNEQY
CL-7445	DRGSQS	YSNGDKE	DNPRGGM	QGHRA	VHGEER	CASSPWDSPNEQY
CL-7467	DRGSQS	YQAGDKE	GNAQGGM	PGHRA	VHGEER	CASSPWDSPNVQY
CL-7475	DRGSQS	YPQGDKK	DNPAGGI	SGHRS	VHGEER	CASSPWDSPNVQY
CL-7480	DRGSQS	YQEGDKE	SNFGNEK	PGHRA	VHGEER	CASSPWDSPNEQY
CL-11581	DRGSQS	YSQGDKE	DNPRGGM	PGHRS	VHGEER	CASSPWDSPNVQY
CL-11594	DRGSQS	YSNGDKE	DNEQGGM	PGHRS	VHGEER	CASSPWDSPNVQY
CL-11611	DRGSQS	YQEGDKE	NNPSGGM	QGHRA	VHGEER	CASSPWDSPNEQY
CL-11614	DRGSQS	YSQGDKE	DNPAGGI	SGHRS	VHGEER	CASSPWDSPNEQY
CL-11623	DRGSQS	YSQGDKE	NNPSGGM	PGHRS	VHGEER	CASSPWDSPNVQY

**Table 4:** PRAME pHLA binding affinity of selected soluble TCER candidates in comparison to the wild type CDR-harboring parental TCR expressed as a single chain TCR construct.

Clone	K_D_ [nM]
CL-7467	3.4
CL-7445	3.7
CL-11581	5.2
CL-11594	6.1
CL-11623	6.6
CL-11611	12.0
CL-7435	16.5
CL-11614	17.8
CL-7480	24.5
CL-7475	37.4
Parental TCR	1230.0

**Table 5:** EC50 values based on cytotoxicity data from selected candidates.

Name	EC_50_ UACC-257 [pM]	EC_50_ HS695T [pM]
CL-7435	1136	1380
CL-7445	115	644
CL-7467	61	172
CL-7475	1864	1556
CL-7480	730	1849
CL-11581	310	587
CL-11594	450	808
CL-11611	656	984
CL-11614	914	1424
CL-11623	136	849

## Data Availability

The data presented in this study are available in this article.
